# Syringomatous carcinoma: case report of a rare tumor entity

**Published:** 2012-07-18

**Authors:** Basma El khannoussi, Hajar Hechlaf, Issam Lalya, Mohamed Oukabli, Abderrahman Al Bouzidi, Nicolas Ortonne

**Affiliations:** 1 Department of Pathology, National Institute of Oncology, Rabat, Morocco; 2 Department of Radiotherapy, National Institute of Oncology, Rabat, Morocco; 3 Department of Pathology, Military Instruction Hospital Mohammed V Rabat, Morocco; 4 Department of Pathology Henri Mondor Hospital, Créteil, France

**Keywords:** Syringomatous carcinoma, histopathology, immunohistochemistry, differential diagnosis

## Abstract

Syringomatous carcinoma is a rare cutaneous neoplasm, most frequently situated on the face and scalp and histologically characterised by an infiltrative pattern of basaloid or squamous cells, a desmoplastic stromal reaction and keratin filled cysts. We report the case of a 76-year-old woman who presented an ulcerative interscapular lesion measuring 3x4cm. After resection, the histological examinations of the specimens have identified a basal cell carcinoma. However, a local recurrence was observed 18 months later; histopathological findings showed a syringomatous pattern and neoplastic epithelial cells arranged in interconnecting cords with microcystic areas. Nests, cords, and tubules of the tumour extended into the dermis and into the adjacent muscle. Sclerosis of stroma around the cords was present. Tumour cells were not connected to the epidermis. The immunohistochemical analysis showed positivity for anti-CK7, AE1/AE3 and negativity for anti CEA and anti CK20. These histological and immunohistochemical analyses were consistent with the diagnosis of syringomatous eccrine carcinoma. Syringomatous carcinoma is an extremely invasive tumor, locally destructive and slowly growing adnexal tumour, derived from eccrine sweat glands. It is often mistaken, both clinically and microscopically, for other benign and malignant entities. The tumour recurrence is high due to extensive perineural invasion, but regional or distant metastases are rare. The local aggressive nature of the tumour and the high recurrence rate may necessitate mutilating procedures. Optimal treatment consists of a complete microscopically controlled surgical excision with clear surgical margins.

## Background

Syringomatous carcinoma (SC) is a rare cutaneous neoplasm, most frequently situated on the face and scalp and histologically characterised by an infiltrative pattern of basaloid or squamous cells, a desmoplastic stromal reaction, keratin filled cysts. This tumor is characterised by cutaneous involvement, invasive destructive local growth, skeletal muscle, and perineural invasion. We describe a patient with a syringomatous carcinoma of the scapular region, demonstrating the local aggressive nature of this tumour. The purpose of this study was to describe the clinical features, the histopathological findings, the treatment, and the difficulties encountered in diagnosing this tumour.

## Patient and observation

A 76-year old woman presented with a 24-month history of enlarging mass involving the back history of trauma. Physical examination showed a mass of an 3x4 cm in diameter, localized in the right inter-scapular region. The mass was ulcerative helophytic, grayish in colour, hard in consistency and easily bleeding on manipulation. The remainder of the examination was unremarkable; no lymphadenopathy and no abdominal masses were felt. After resection, the histological examinations of the specimens have concluded for basal cell carcinoma.

**Figure 1:**
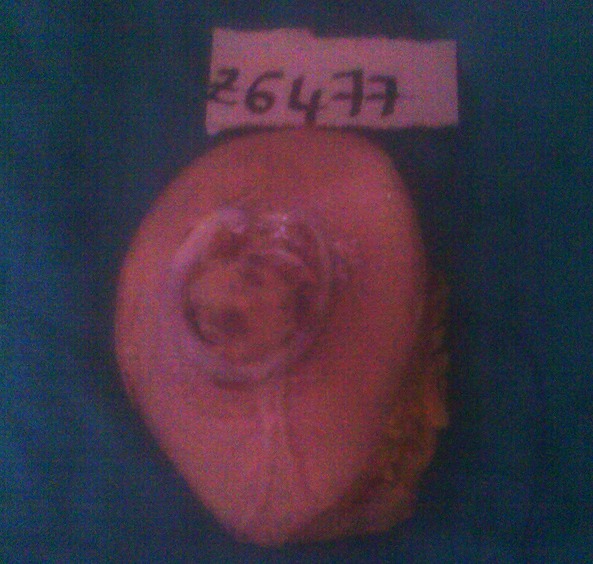
Surgical excision showing an ulcerative nodular lesion hard in consistency

**Figure 2:**
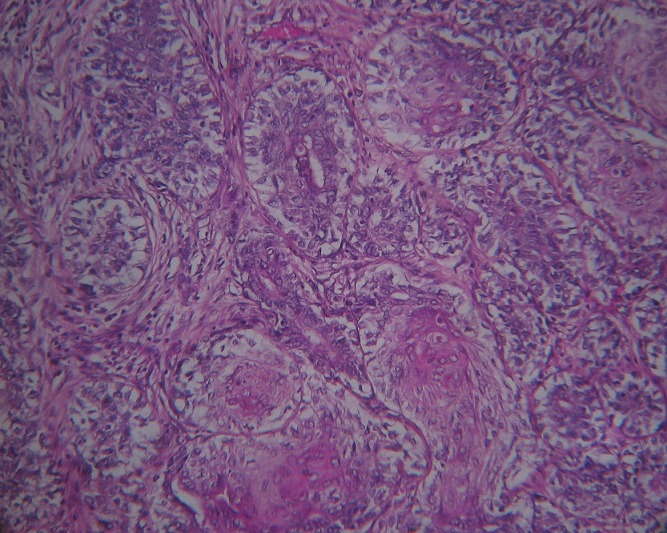
Infiltration of the dermis by tubules and cords (sudoral differentiation) with focal squamous differentiation (HS magnification 10)

**Figure 3:**
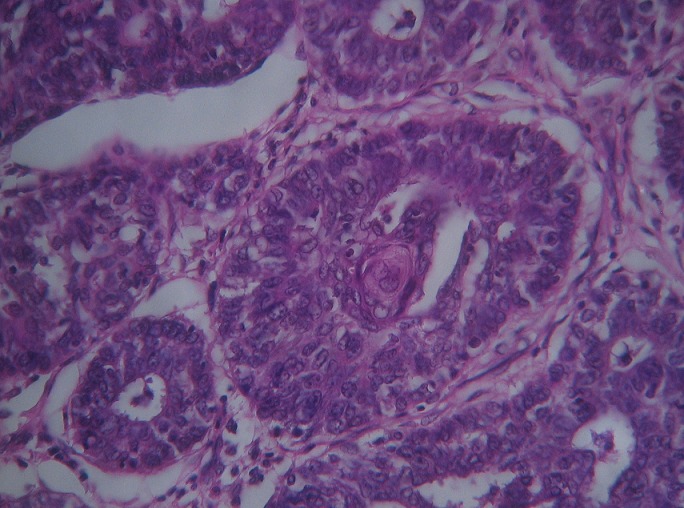
Infiltration of the dermis by tubules and cords (sudoral differentiation) with focal squamous differentiation (HS magnification 20)

**Figure 4:**
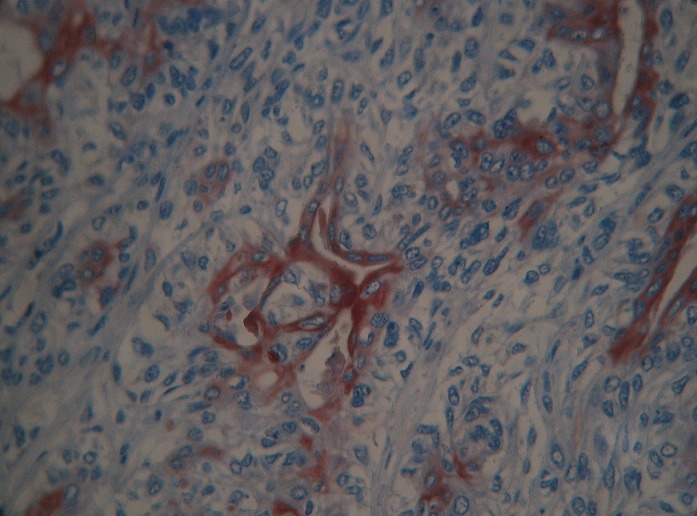
Expression of cytokératine 7 (Avidine Biotine magnification 40)

A local recurrence was observed 18 months later; the patient was admitted to our institution for Lumpectomy (Figure 1). Histopathological examination revealed a syringomatous pattern infiltrating the dermis (Figure 2, Figure 3), subcutis and skeletal muscle. The neoplastic epithelial cells were arranged in interconnecting cords with microcystic areas. Nests, cords, and tubules of the tumour extended into the dermis and into the adjacent muscle. Many lobules showed squamous differentiation. Sclerosis of stroma around the cords was present. Tumour cells were not connected to the epidermis. The immunohistochemical analysis showed positivity for anti-CK7 (Figure 4), AE1/AE3 and negativity for anti CEA and anti CK20. Based upon her histological and immunohistochemical presentation, the diagnosis of syringomatous eccrine carcinoma was established. Radiotherapy of the involved area was performed (70 Gy, 35 sessions)

## Discussion

Syringomateux carcinoma (SC) is a rare malignant skin appendageal tumor deriving from sweat glands, first described by Goldstein et al in 1982 as a microcystic adnexal carcinoma [1, 2]. Since 1970, a variety of synonyms have been given to this carcinoma (Syringoid eccrine carcinoma, microcystic adnexal carcinoma, malignant syringoma, syringomateux carcinoma, adnexal carcinoma with appearance syringomateux) [3]. It is currently believed that these are different names for the same neoplastic process with different degrees of differentiation.

Most series show a median age of presentation in the fourth and fifth decade (age range of 20s to 80s) with equal frequency in the two sexes [4, 5]. The most common sites of involvement are the head and neck regions with a predilection for centrofacial region (about 85% of cases). Less frequent sites include axilla, trunk and extremities [5]. The clinical signs of SC include a subcutaneous indurated nodule or insulated plate with pink or yellow coloured, ill defined margins and overlying telangiectasia. The epidermis may appear normal, atrophic of scaly. Ulceration is unusual [6]. Our patient had a large and extensively ulcerated tumor.

The histological appearance includes formation of tubules, small elongated nests, cords and cysts. These structures are lined with a double layer of cells with flattened cuboidal or squamous differentiation. Cytonuclear atypia and mitosis are generally mild to moderate. Perineural and intraneural invasion is commun and a particularly characteristic finding in SC [4]. This tumor extend deep into the dermis, into subcutaneous and fat, into skeletal muscle and sometimes into bone in an abundant desmoplastic and fibrous stroma [5]. The invasive tumor is locally destructive but lymph node and distant metastasis are rare. The tumor cells express typically cytokeratin AE1/AE3, MEA, CAM 5-2, Leu M1, CEA and PS100 [1, 7]. The histological diagnosis of this tumor is difficult to bear especially in front biopsy of small size or superficial or when the characteristic histological features may not be apparent [6]. The differential diagnosis mainly concerns syringoma, desmoplastic trichoepithelioma, squamous cell carcinoma, basal sclerodermiform cell carcinoma, trichoadénoma, cutaneous metastasis of breast carcinoma. In our case, the architectural pattern is indicative of malignant neoplasm but ductal differentiation is overlooked, an erroneous diagnosis of morphea-like basal cell or squamous carcinoma is made.

There are a few publications regarding the characteristics, treatment options, and outcome of patients with SC. Complete surgical excision is the method of choice for treatment , in the literature radiation therapy is rarely applied because this tumor still radio resistant. Lymphatic dissemination and metastases are rare [5, 8, 9], but tumor recurrence are frequent. In the literature, about 40 to 60% of patients had one or more local recurrences within 6 months to 30 years after treatment with standard wide local excision [6].

## Conclusion

Syringomatous carcinoma is extremely invasive tumor, locally destructive, slowly growing adnexal tumour, derived from eccrine sweat glands. It is often mistaken, both clinically and microscopically, for other benign and malignant entities. The tumour recurrence is high due to extensive perineural invasion, but regional or distant metastases are rare. The local aggressive nature of the tumour and the high recurrence rate may necessitate mutilating procedures. Optimal treatment consists of a complete microscopically controlled surgical excision with clear surgical margins.
